# How Do the Four Core Factors of High Entropy Affect the Electrochemical Properties of Energy‐Storage Materials?

**DOI:** 10.1002/advs.202411291

**Published:** 2024-11-04

**Authors:** Wenze Wang, Qian Zhang, Liting Yang, Guisheng Liang, Xuhui Xiong, Yifeng Cheng, Limin Wu, Chunfu Lin, Renchao Che

**Affiliations:** ^1^ College of Physics Donghua University Shanghai 201620 China; ^2^ Laboratory of Advanced Materials Shanghai Key Lab of Molecular Catalysis and Innovative Materials Academy for Engineering & Technology Advanced Coatings Research Center of Ministry of Education of China Fudan University Shanghai 200438 China; ^3^ Institute of Materials for Energy and Environment School of Materials Science and Engineering Qingdao University Qingdao 266071 China; ^4^ Zhejiang Laboratory Hangzhou 311100 China; ^5^ Inner Mongolia University Hohhot 010021 China

**Keywords:** electrochemical property, four core factors of high entropy, high‐entropy material, high‐temperature operation, in situ characterization

## Abstract

High‐entropy materials (HEMs) are extremely popular for electrochemical energy storage nowadays. However, the detailed effects of four core factors of high entropy on the electrochemical properties of HEMs are still unclear. Here, a high‐entropy La_1/4_Ce_1/4_Pr_1/4_Nd_1/4_Nb_3_O_9_ (HE‐LaNb_3_O_9_) oxide is prepared through multiple rare‐metal‐ion substitution in LaNb_3_O_9_, and uses HE‐LaNb_3_O_9_ as a model material to systematically study the effects of the four core factors of high entropy on electrochemical energy‐storage materials. The high‐entropy effect lowers the calcination temperature for obtaining pure HE‐LaNb_3_O_9_. The lattice distortion in HE‐LaNb_3_O_9_ leads to its decreased unit‐cell‐volume variations, which benefits its cyclability. Based on the restrained diffusion arising from the lattice distortion, the Li^+^ diffusivity of HE‐LaNb_3_O_9_ at room temperature (25 °C) is limited, which causes its lowered rate capability. However, the Li^+^ diffusivity of HE‐LaNb_3_O_9_ at high temperature (60 °C) becomes faster than that of LaNb_3_O_9_, which is attributed to the alleviated lattice distortion at the high‐temperature, resulting in higher rate capability. The cocktail effects in HE‐LaNb_3_O_9_ enable its larger electronic conductivity, better electrochemical activity, more intensive Nb^5+^ ↔ Nb^3+^ redox reaction, and larger reversible capacity. The insight gained here can provide a guide for the rational design of new HEMs with good energy‐storage properties.

## Graphical abstract

High‐entropy La_1/4_Ce_1/4_Pr_1/4_Nd_1/4_Nb_3_O_9_ is selected as a model material to systematically study the effects of the four core factors of high entropy (i.e., high‐entropy effects, lattice distortion, restrained diffusion, and cocktail effects) on the electrochemical properties (i.e., reversible capacity, rate capability, cyclability, and high‐temperature performance) of energy‐storage materials. The insight gained here provides a guide for rationally enhancing the energy‐storage properties.

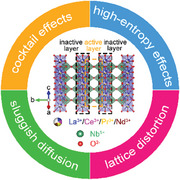

## 1 Introduction

High‐entropy materials (HEMs) have attracted intensive attention due to their tailorable and unexpected properties. The high‐entropy concept aims to introduce various elements on a single crystallographic site that can stabilize the solid‐solution state, leading to high configurational entropy (Δ*S*_config_ > 1.5*R*).^[^ ^1^ ^]^ This interesting concept was first applied to alloy systems.^[^ ^2^ ^]^ Yeh summarized four core factors affecting the microstructures and properties of high‐entropy alloys (HEAs)^[^ ^3^ ^]^: 1) high‐entropy effects, 2) lattice distortion, 3) restrained diffusion, and 4) cocktail effects. The high‐entropy effect is beneficial for decreasing the formation temperature of HEAs. The lattice distortion in HEAs caused by the size differences of the different ions at the same crystallographic site increases the strength and hardness of HEAs. The resulting restrained diffusion limits the ion diffusivity in HEAs. The cocktail effects affect the properties of HEAs through the element interaction, and HEAs can be customized with the required properties by changing the stoichiometry and element type.

Recently, the high‐entropy concept has been extended to several non‐metallic materials, including oxides,^[^ ^4^ ^]^ borides,^[^ ^5^ ^]^ carbides,^[^ ^6^ ^]^ nitrides,^[^ ^7^ ^]^ fluorides,^[^ ^8^ ^]^ sulfides,^[^ ^9^ ^]^ etc. For instance, Sarkar et al.^[^ ^10^ ^]^ explored a rock‐salt (Co_0.2_Cu_0.2_Mg_0.2_Ni_0.2_Zn_0.2_)O oxide as the first high‐entropy electrode material for Li^+^‐storage application. Its excellent cyclability was attributed to configurational entropy stabilization, which conserved the original rock‐salt crystal structure. In addition, the Li^+^‐storage properties of the active material are tailored by modifying the composition. Yan et al.^[^ ^11^ ^]^ studied a [(Bi,Na)_1/5_(La,Li)_1/5_(Ce,K)_1/5_Ca_1/5_Sr_1/5_]TiO_3_ perovskite anode material with stable performance, ascribed to the charge compensation mechanism and unique entropy‐stabilized structure. Clearly, HEMs are very promising for electrochemical energy storage. However, the effects of the four core factors of high entropy on the electrochemical properties of HEMs have not been systematically studied, and thus need to be clarified in order to rationally design new HEMs with better energy‐storage properties.

Here, high‐entropy La_1/4_Ce_1/4_Pr_1/4_Nd_1/4_Nb_3_O_9_ (HE‐LaNb_3_O_9_) with a configurational entropy of 1.56*R*, prepared through substituting different rare‐metal ions (Ce^3+^, Pr^3+^, and Nd^3+^) for 75% of La^3+^ in LaNb_3_O_9_, is selected as a model material to systematically study the effects of the four core factors of high entropy on electrochemical energy‐storage materials. HE‐LaNb_3_O_9_ is a high‐entropy material suitable for this study because the very similar ionic radii of La^3+^ (0.136 nm), Ce^3+^ (0.134 nm), Pr^3+^ (≈0.130 nm), and Nd^3+^ (0.127 nm) result in minor influences on the lattice constants.^[^ ^12^ ^]^ 1) Based on the high‐entropy effects, pure HE‐LaNb_3_O_9_ is obtained at a calcination temperature obviously lower than that of LaNb_3_O_9_. 2) The lattice distortion in HE‐LaNb_3_O_9_ significantly inhibits its unit‐cell‐volume variations during lithiation/delithiation, leading to its enhanced cyclability. 3) The restrained diffusion limits the Li^+^ diffusivity of HE‐LaNb_3_O_9_ at room temperature, causing its lower rate capability than that of LaNb_3_O_9_. However, the alleviated lattice distortion in HE‐LaNb_3_O_9_ at high temperature allows its faster Li^+^ diffusivity and higher rate capability than that of LaNb_3_O_9_. 4) The cocktail effects in HE‐LaNb_3_O_9_ enable its significantly higher electronic conductivity, better electrochemical activity, more intensive Nb^5+^ ↔ Nb^3+^ redox reaction, and larger reversible capacity.

## 2 Results and Discussion

### 2.1 Structure and Phase Characterizations

**Figure 1:**
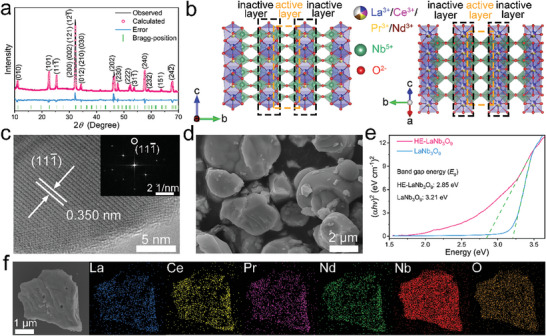
Physico‐chemical characterizations of HE‐LaNb_3_O_9_. a) Rietveld‐refined XRD pattern (main peaks are labeled). b) Schematic crystal structure. c) HRTEM image (inset: FFT pattern). d) FESEM image. e) Evolution of optical bandgaps based on UV–vis absorption spectra. f) EDS mapping images.

The Rietveld refinement based on the powder X‐ray diffraction (XRD) pattern of HE‐LaNb_3_O_9_ (**Figure** **1**a) suggests a monoclinic structure with a *P2/m* space group, which is the same as that of LaNb_3_O_9_ (Figure S1, Supporting Information), indicating that the crystal structure does not change after the high‐entropy modification. HE‐LaNb_3_O_9_ owns a cation‐deficient perovskite crystal structure (Figure 1b; Figure S2, Supporting Information). All Nb^5+^ ions are located at 4*o* sites, connecting with the surrounding O^2−^ ions to form electrochemical active NbO_6_ octahedra. All 2*m* dodecahedra are inactive, and randomly filled by vacancies, La^3+^, Ce^3+^, Pr^3+^, and Nd^3+^ ions in a molar ratio of 1:1/2:1/2:1/2:1/2, generating a high configurational entropy of 1.56*R* (Equation S1, Supporting Information). In contrast, all the 2*m* dodecahedra of LaNb_3_O_9_ are randomly filled by vacancies and La^3+^ ions in a molar ratio of 1:2. The accurate fractional atomic constants of HE‐LaNb_3_O_9_ and LaNb_3_O_9_ are listed in Tables S1 and S2 (Supporting Information), respectively. The lattice constants of HE‐LaNb_3_O_9_ (*a* = 0.552149(21) nm, *b* = 0.786961(22) nm, *c* = 0.551657(21) nm, *β* = 90.175(3)^°^, and *V* = 0.239703(15) nm^3^) are slightly smaller than that of LaNb_3_O_9_ (*a* = 0.553915(13) nm, *b* = 0.791256(14) nm, *c* = 0.553738(13) nm, *β* = 90.229(2)^°^, and *V* = 0.242697(9) nm^3^). These tiny lattice‐constant differences are attributed to the fact that Ce^3+^ (0.134 nm), Pr^3+^ (≈0.130 nm), and Nd^3+^ (0.127 nm) have slightly smaller ionic radii than La^3+^ (0.136 nm).^[^ ^12^ ^]^ After the calcination at 1200 °C, pure HE‐LaNb_3_O_9_ is obtained, whereas impurities appear in LaNb_3_O_9_ (Figure S3, Supporting Information), indicating that the high‐entropy modification decreases the calcination temperature of LaNb_3_O_9_. When carefully comparing the XRD patterns of HE‐LaNb_3_O_9_ and LaNb_3_O_9_ obtained at the same high calcination temperature of 1300 °C, it is found that the peak width of HE‐LaNb_3_O_9_ is significantly wider than that of LaNb_3_O_9_ (Figure S4, Supporting Information), which suggests that the high‐entropy modification induces severe lattice distortion.

The monoclinic crystal structure and high crystallinity of HE‐LaNb_3_O_9_ are confirmed by the fast Fourier transform (FFT) pattern and high‐resolution transmission electron microscopy (HRTEM) image (Figure 1c). The field emission scanning electron microscopy (FESEM) and transmission electron microscopy (TEM) images (Figure 1d; Figures S5 and S6, Supporting Information) show that the morphology (irregular particles) and particle size (1–5 µm) of HE‐LaNb_3_O_9_ are similar to that of LaNb_3_O_9_. The Branauer–Emmett–Teller (BET) specific surface area is determined to be a small value of 0.67 m^2^ g^−1^, which is comparable to that of LaNb_3_O_9_ (0.62 m^2^ g^−1^, Figure S7, Supporting Information). Therefore, the high‐entropy modification negligibly affects the morphology and size of LaNb_3_O_9_. However, the bandgap of HE‐LaNb_3_O_9_ (2.85 eV) revealed by the UV–vis absorption spectrum (Figure 1e; Figure S8 and Equation S2, Supporting Information) is significantly smaller than that of LaNb_3_O_9_ (3.21 eV), suggesting the significantly larger electronic conductivity of HE‐LaNb_3_O_9_. The tested electronic conductivity of HE‐LaNb_3_O_9_ (3.6 × 10^−7^ S cm^−1^), in fact, is two orders of magnitude larger than that of LaNb_3_O_9_ (8.5 × 10^−9^ S cm^−1^). This electronic‐conductivity improvement is undoubtedly ascribed to the cocktail effects after the high‐entropy modification, in which the free electrons in Ce^3+^, Pr^3+^, and Nd^3+^ facilitate electron transport within HE‐LaNb_3_O_9_. Uniform La, Ce, Pr, Nd, Nb, and O distributions are demonstrated in the energy dispersive spectroscopy (EDS) mapping images (Figure 1f), confirming the high entropy and purity of HE‐LaNb_3_O_9_. The molar ratio of La:Ce:Pr:Nd:Nb in HE‐LaNb_3_O_9_ obtained by an inductively coupled plasma atomic emission spectrometer (ICP‐AES) experiments is 0.24:0.26:0.25:0.24:3, which is well consistent with its theoretical ratio (1/4:1/4:1/4:1/4:3).

### 2.2 Redox Mechanisms

**Figure 2:**
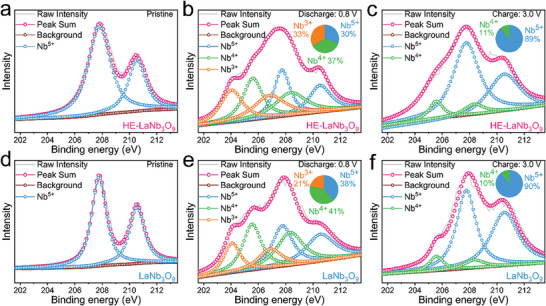
Redox mechanisms of HE‐LaNb_3_O_9_ compared with LaNb_3_O_9_. Ex situ Nb‐3*d* XPS spectra of HE‐LaNb_3_O_9_: a) pristine, b) discharge (0.8 V), and c) charge (3.0 V) states. Ex situ Nb‐3*d* XPS spectra of LaNb_3_O_9_: d) pristine, e) discharge (0.8 V), and f) charge (3.0 V) states.

The X‐ray photoelectron spectroscopy (XPS) survey spectrum of HE‐LaNb_3_O_9_ reveals the presence of not only the original elements (La, Nb, and O) but also the high‐entropy substitution elements (Ce, Pr, and Nd), which can be regarded as another proof of the successful co‐substitution of the Ce, Pr, and Nd elements into the La sites in the LaNb_3_O_9_ lattice (Figure S9, Supporting Information). The Nb‐3*d* XPS spectrum of HE‐LaNb_3_O_9_ exhibits peaks at 207.8 and 210.6 eV (**Figure** **2**a), respectively attributed to the Nb‐3*d*_5/2_ and Nb‐3*d*_3/2_ of Nb^5+^.^[^ ^13^ ^]^ After discharge to 0.8 V (Figure 2b), the Nb‐3*d* XPS spectrum matches well with a combination of Nb^5+^ (30%), Nb^4+^ (37%), and Nb^3+^ (33%), and the Nb ions after charge to 3.0 V (Figure 2c) are restored to an appropriate valence containing 89% of Nb^5+^ and 11% of Nb^4+^.^[^ ^14^ ^]^ Compared with HE‐LaNb_3_O_9_, LaNb_3_O_9_ does not show changes in the peak positions of Nb‐3*d*_5/2_ and Nb‐3*d*_3/2_ (Figure 2d), which indicates that the high‐entropy modification does not change the Nb valence. A higher Nb valence (38% of Nb^5+^, 41% of Nb^4+^, and 21% of Nb^3+^) is obtained after discharge to 0.8 V (Figure 2e) and the Nb ions after charge to 3.0 V (Figure 2f) are restored to a similar valence (90% of Nb^5+^ and 10% of Nb^4+^) compared with that of HE‐LaNb_3_O_9_, revealing that the Nb^5+^ ↔ Nb^3+^ redox mechanism does not change but the reversible capacity is increased after the high‐entropy modification.

**Figure 3:**
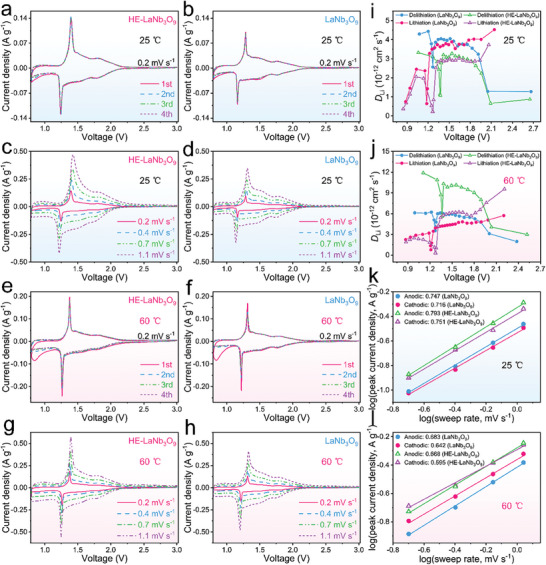
CV tests and electrochemical kinetics of HE‐LaNb_3_O_9_ compared with that of LaNb_3_O_9_ at 25 and 60 °C. CV curves of a) HE‐LaNb_3_O_9_/Li and b) LaNb_3_O_9_/Li half cells at 0.2 mV s^−1^ and 25 °C. CV curves of c) HE‐LaNb_3_O_9_/Li and d) LaNb_3_O_9_/Li half cells at different sweep rates and 25 °C. CV curves of e) HE‐LaNb_3_O_9_/Li and f) LaNb_3_O_9_/Li half cells at 0.2 mV s^−1^ and 60 °C. CV curves of g) HE‐LaNb_3_O_9_/Li and h) LaNb_3_O_9_/Li half cells at different sweep rates and 60 °C. Variations in apparent Li^+^ diffusion coefficients of HE‐LaNb_3_O_9_ compared with LaNb_3_O_9_ during lithiation–delithiation at i) 25 and j) 60 °C. Calculation of *b*‐values for HE‐LaNb_3_O_9_/Li half cell compared with LaNb_3_O_9_/Li half cell by using relationship between peak current and sweep rate at k) 25 and l) 60 °C.

The initial four‐cycle cyclic voltammetry (CV) curves of the HE‐LaNb_3_O_9_/Li half cell at 0.2 mV s^−1^ and 25 °C (**Figure** **3**a) show a couple of sharp reduction/oxidation peaks at 1.78/1.80 and 1.24/1.38 V, which can be assigned to the Nb^5+^/Nb^4+^ and Nb^4+^/Nb^3+^ redox couples, respectively.^[^ ^15^ ^]^ The almost overlap of these CV curves indicates the excellent electrochemical stability of HE‐LaNb_3_O_9_ even during the initial activation process. The CV curve shape and peak position of LaNb_3_O_9_ (Figure 3b) are similar to that of HE‐LaNb_3_O_9_, which confirms that the high‐entropy modification does not affect the redox mechanism of LaNb_3_O_9_. With increasing the sweep rate, the peak intensities of HE‐LaNb_3_O_9_ (Figure 3c) are always significantly larger than that of LaNb_3_O_9_ (Figure 3d), suggesting the better electrochemical activity of HE‐LaNb_3_O_9_. This improvement can be due to the multi‐ion interaction (cocktail effects) promoting the Nb^5+^ ↔ Nb^3+^ redox reaction in HE‐LaNb_3_O_9_.

At 60 °C, the initial CV cycles of HE‐LaNb_3_O_9_ and LaNb_3_O_9_ are obviously different from their following three overlapped cycles (Figure 3e,f), which is attributed to the generation of thicker solid‐electrolyte interphase (SEI) films during the activation process (Figure S10, Supporting Information).^[^ ^16^ ^]^ However, the curve misalignment of HE‐LaNb_3_O_9_ in the first CV cycle is less obvious than that of LaNb_3_O_9_ (Figure 3f), indicating that the SEI film in HE‐LaNb_3_O_9_ (Figure S10c, Supporting Information) is thinner than that in LaNb_3_O_9_ (Figure S10d, Supporting Information). At the high temperature, the electrochemical activity is enhanced, as indicated by the increased peak intensities (Figure 3g), and HE‐LaNb_3_O_9_ still exhibits better electrochemical activity than LaNb_3_O_9_ (Figure 3h).

### 2.3 Li^+^ Diffusivity and Kinetics Analyses

According to Fick's second law, the Li^+^ diffusion coefficients (*D*_Li_) of HE‐LaNb_3_O_9_ and LaNb_3_O_9_ are calculated on basis of the galvanostatic intermittent titration technique (GITT) curves (Figure 3i,j; Figure S11 and calculation details in Supporting Information).^[^ ^17^ ^]^ The average *D*_Li_ value of HE‐LaNb_3_O_9_ is 2.35 × 10^−12^ cm^2^ s^−1^ at 25 °C, smaller than that of LaNb_3_O_9_ (3.36 × 10^−12^ cm^2^ s^−1^), which can be mainly explained by the restrained diffusion arising from the lattice distortion. At 60 °C, the average *D*_Li_ value of HE‐LaNb_3_O_9_ is increased to 6.63 × 10^−12^ cm^2^ s^−1^, surpassing not only that at 25 °C but also that of LaNb_3_O_9_ (4.53 × 10^−12^ cm^2^ s^−1^) at 60 °C. Therefore, the lattice distortion of HE‐LaNb_3_O_9_ is significantly alleviated at the high‐temperature.

**Figure 4:**
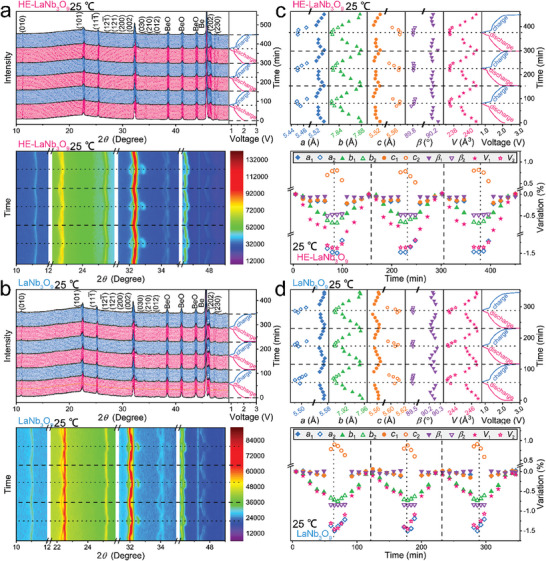
Crystal‐structure evolutions of HE‐LaNb_3_O_9_ compared with that of LaNb_3_O_9_ at 25 °C. Original and contour in situ XRD patterns of a) HE‐LaNb_3_O_9_/Li and b) LaNb_3_O_9_/Li in situ cells with corresponding GCD curves (initial three cycles). Variations in lattice constants of c) HE‐LaNb_3_O_9_ and d) LaNb_3_O_9_ (initial three cycles).

The equation of *I* = *av^b^* is employed to study the intercalation‐pseudocapacitive behavior of HE‐LaNb_3_O_9_ and LaNb_3_O_9_, in which *I* is the peak current, *v* is the sweep rate, and *a* and *b* are adjustable constants.^[^ ^18^ ^]^ The cases of *b* = 1 and *b* = 0.5 respectively indicate that lithiation and delithiation are totally controlled by capacitive behavior and diffusion behavior. At 25 °C, the *b* values for HE‐LaNb_3_O_9_ are 0.751 and 0.793, respectively, which are larger than that of LaNb_3_O_9_ (0.716 and 0.747, **Figure** **4**k). Although the Li^+^ diffusion of HE‐LaNb_3_O_9_ is lowered after the high‐entropy modification, its fast intercalation‐pseudocapacitive behavior enhanced by the multi‐ion interaction undoubtedly facilitates the electrochemical‐property (especially rate‐capability) improvement. At 60 °C, the *b* values for HE‐LaNb_3_O_9_ are decreased to 0.595 and 0.668 (Figure 4l), which can be due to the faster Li^+^ diffusivity at the higher temperature. Importantly, these two *b* values for HE‐LaNb_3_O_9_ are smaller than that for LaNb_3_O_9_ (0.642 and 0.683), undoubtedly rooted in its weakened lattice distortion at the high‐temperature.

### 2.4 In Situ XRD Characterizations

The HE‐LaNb_3_O_9_/Li in situ cell delivers a discharge capacity of 131.2 mAh g^−1^ at 0.4C during the first cycle. Although HE‐LaNb_3_O_9_ undergoes a two‐phase transformation at a voltage of ≈1.3 V, the phase after the phase transformation remains the *P2/m* space group symmetry. From the open circuit voltage (OCV) to ≈1.3 V during the initial discharging process, the most intensive peak, which is superposed by the (12$\bar{1}$), (121), (200), and (002) peaks, first move toward higher angles, then slightly toward lower angles, and finally toward slightly higher angles; the (030), (210), and (012) peaks continuously move toward higher angles. From ≈1.3 V to 0.8 V, the (12$\bar{1}$), (121), (200), (030), and (210) peaks continue moving to higher angles, whereas the (002) and (012) peaks progressively move to lower angles. During the charging process, the 2*θ* positions and intensities of all the peaks are almost fully covered. In addition, all these peaks exhibit almost the same evolutions during the subsequent charging–discharging processes, indicating the good crystal‐structure stability of the intercalating HE‐LaNb_3_O_9_ during the first three cycles, which greatly benefits the electrochemical properties (especially the cyclability). LaNb_3_O_9_ also undergoes similar phase transformation, and all XRD diffraction peaks and evolution trends of LaNb_3_O_9_ (Figure 4b) are similar to that of HE‐LaNb_3_O_9_. However, the LaNb_3_O_9_/Li in situ cell shows a small discharge capacity of 90.3 mAh g^−1^.

The Rietveld‐refined lattice constants (*a*, *b*, *c*, *β*, and *V*) of HE‐LaNb_3_O_9_ exhibit complicated variations (Figure 4c), but perfectly match with the above reversible diffraction‐peak evolution. The maximum *V*‐value variation of HE‐LaNb_3_O_9_ is calculated to be −1.36% after lithiation. During the lithiation and delithiation, the NbO_6_ octahedron layers provide electrochemical activity, whereas the inactive (vacancy/La/Ce/Pr/Nd)O_12_ dodecahedron layers own excellent volume‐buffering capability, leading to the small unit‐cell‐volume changes caused by Li^+^ intercalation/de‐intercalation and Nb‐ion reduction/oxidation.^[^ ^19^ ^]^ All the lattice constants of LaNb_3_O_9_ show similar variation trends to that of HE‐LaNb_3_O_9_, but the variations are larger, revealing a maximum *V*‐value variation of −1.47% (Figure 4d). Although more Li^+^ ions insert into HE‐LaNb_3_O_9_ (discharge capacity of 131.2 mAh g^−1^) than LaNb_3_O_9_ (discharge capacity of 90.3 mAh g^−1^), the maximum *V*‐value variation of HE‐LaNb_3_O_9_ is 7.5% smaller. When the same amount of Li^+^ ions are intercalated, HE‐LaNb_3_O_9_ exhibits ≈37% smaller *V*‐value variations than LaNb_3_O_9_. The main reason why the *V*‐value variations of HE‐LaNb_3_O_9_ become smaller after the high‐entropy modification can be that its lattice distortion inhibits its unit‐cell‐volume variations during lithiation/delithiation, which is undoubtedly beneficial for its cyclability.

**Figure 5:**
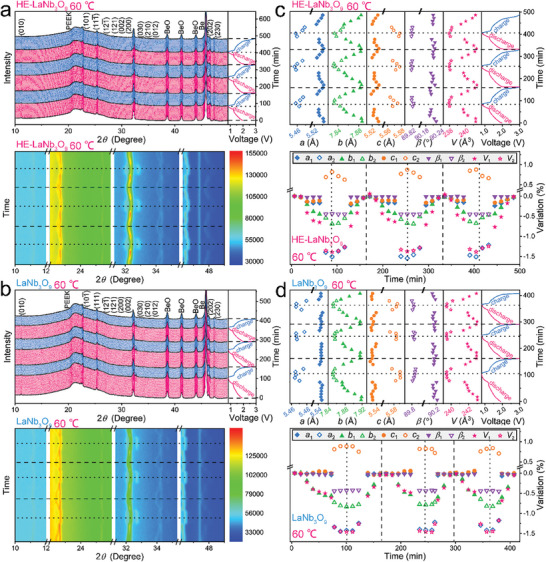
Crystal‐structure evolutions of HE‐LaNb_3_O_9_ compared with that of LaNb_3_O_9_ at 60 °C. Original and contour in situ XRD patterns of a) HE‐LaNb_3_O_9_/Li and b) LaNb_3_O_9_/Li in situ cells with corresponding GCD curves (initial three cycles). Variations in lattice constants of c) HE‐LaNb_3_O_9_ and d) LaNb_3_O_9_ (initial three cycles).

At 60 °C, all the diffraction peaks and lattice constants of HE‐LaNb_3_O_9_ exhibit the same evolution trends as that at 25 °C (**Figure** **5**). However, their variations are slightly larger, which can be due to the reason that the lattice distortion is alleviated at the high temperature. In fact, the widths of the in situ diffraction peaks at 60 °C are narrower than that at 25 °C (Figure S12, Supporting Information), fully demonstrating the alleviated lattice distortion at the high temperature. Importantly, the maximum *V*‐value change of HE‐LaNb_3_O_9_ (−1.38%) at 60 °C is still smaller than that of LaNb_3_O_9_ (−1.45%).

**Figure 6:**
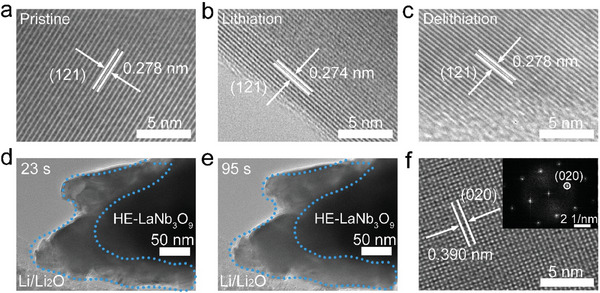
Ex situ and in situ TEM examinations of HE‐LaNb_3_O_9_. Ex situ HRTEM images of HE‐LaNb_3_O_9_ at: a) pristine, b) lithiated (0.8 V), and c) delithiated (3.0 V) states. In situ TEM images of Li^+^ insertion in HE‐LaNb_3_O_9_ at: d) 23 and e) 95 s. f) In situ HRTEM image (inset: in situ FFT pattern) of HE‐LaNb_3_O_9_ recorded at lithiated state.

The lattice‐constant changes of HE‐LaNb_3_O_9_ are confirmed by its ex situ HRTEM characterization, which reveals that the (121) interplanar spacing decreases from 0.278 nm (OCV pristine state, **Figure** **6**a) to 0.274 nm (0.8 V lithiated state, Figure 6b) and recovers 0.278 nm (3.0 V delithiated state, Figure 6c). Meanwhile, in situ TEM is employed to further study the particle‐volume change and crystal‐structure evolution of HE‐LaNb_3_O_9_ during lithiation.^[^ ^13^ ^]^ As the in situ bias increases, Li^+^ ions extract from the lithium metal, then pass through the Li_2_O solid electrolyte, and finally insert into the HE‐LaNb_3_O_9_ particle. The strain fringes arising from the Li^+^ insertion and phase transformation fast move. However, the morphology and volume of the particle do not obviously change (Figure 6d,e; Video S1, Supporting Information). Additionally, the in situ FFT pattern and the in situ HRTEM image after lithiation show little change (Figure 6f), further confirming the slight lattice‐constant changes. These in situ TEM results are consistent with the above‐mentioned in situ XRD characterizations.

### 2.5 Li^+^‐Storage Properties

**Figure 7:**
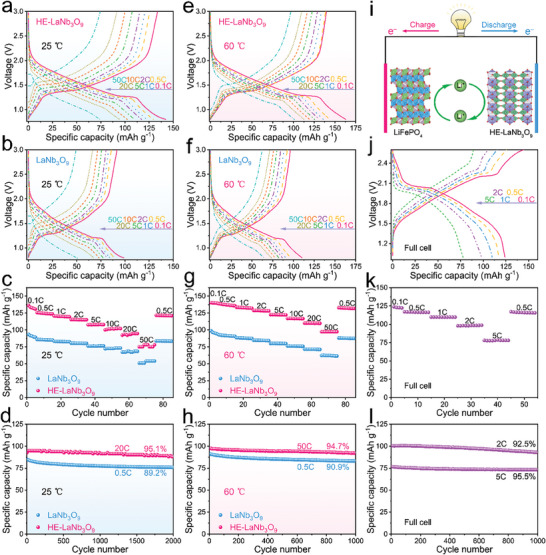
Electrochemical properties of HE‐LaNb_3_O_9_ compared with that of LaNb_3_O_9_ at 25 and 60 °C. GCD curves of a) HE‐LaNb_3_O_9_/Li and b) LaNb_3_O_9_/Li half cells at different current rates, c) rate capability of HE‐LaNb_3_O_9_/Li half cell compared with that of LaNb_3_O_9_/Li half cell, d) cyclability of HE‐LaNb_3_O_9_/Li half cell compared with that of LaNb_3_O_9_/Li half cell at 25 °C. GCD curves of e) HE‐LaNb_3_O_9_/Li and f) LaNb_3_O_9_/Li half cells at different current rates, g) rate capability of HE‐LaNb_3_O_9_/Li half cell compared with that of LaNb_3_O_9_/Li half cell, h) cyclability of HE‐LaNb_3_O_9_/Li half cell compared with that of LaNb_3_O_9_/Li half cell at 60 °C. i) Schematic diagram of LiFePO_4_/HE‐LaNb_3_O_9_ full cell, j) GCD curves of LiFePO_4_/HE‐LaNb_3_O_9_ full cell at different current rates, k) rate capability of LiFePO_4_/HE‐LaNb_3_O_9_ full cell, l) cyclability of LiFePO_4_/HE‐LaNb_3_O_9_ full cell at 25 °C.

The discharge curve of HE‐LaNb_3_O_9_ fast drops from 3.0 to 2.1 V, slowly drops to 1.3 V, stabilizes at 1.3–1.2 V, and finally fast drops to 0.8 V (**Figure** **7**a; Figure S13a, Supporting Information), which is similar to that of LaNb_3_O_9_ (Figure 7b; Figure S13b, Supporting Information). In the initial cycle, the reversible capacity of HE‐LaNb_3_O_9_ reaches 136.2 mAh g^−1^ with an initial Coulombic efficiency of 81.6% and average operating potential of ≈1.46 V. This capacity is much larger than that of LaNb_3_O_9_ (93.4 mAh g^−1^), which is attributed to the more intensive Nb^5+^ ↔ Nb^3+^ redox reaction in HE‐LaNb_3_O_9_ rooted in its cocktail effects. HE‐LaNb_3_O_9_ remains reversible capacities of 126.1, 120.6, 115.3, 107.6, 100.1, 93.3, and 75.1 mAh g^−1^ at 0.5, 1, 2, 5, 10, 20, and 50C, respectively, which are always larger than that of LaNb_3_O_9_ (Figure 7c). However, when increasing the current rate from 0.1 to 50C, the capacity ratio of HE‐LaNb_3_O_9_ (55.2%) is slightly smaller than that of LaNb_3_O_9_ (58.1%), which can be ascribed to the slower Li^+^ diffusivity of HE‐LaNb_3_O_9_. Furthermore, HE‐LaNb_3_O_9_ shows excellent cyclability with 95.1% capacity retention at 20C after 2000 cycles with no particle crack (Figure S14, Supporting Information), whereas the capacity retention of LaNb_3_O_9_ is only 89.2% at 0.5C after 2000 cycles (Figure 7d). It is well known that a larger reversible capacity causes lower capacity retention. Although HE‐LaNb_3_O_9_ delivers a larger initial reversible capacity for the cycling (93.5 mAh g^−1^ for HE‐LaNb_3_O_9_ vs 84.6 mAh g^−1^ for LaNb_3_O_9_), its capacity retention is higher, fully demonstrating its better cyclability, which can be well explained by its smaller maximum unit‐cell‐volume variation during lithiation/delithiation. When large active material content (80 wt%) and active‐material loading (>6 mg cm^−2^) are used, HE‐LaNb_3_O_9_ also exhibits lower rate capability and better cyclability than LaNb_3_O_9_ (Figure S15, Supporting Information).

At 60 °C, the galvanostatic charge–discharge (GCD) curve shape and the average operating potential of HE‐LaNb_3_O_9_ (≈1.50 V) are very similar to that at 25 °C (Figure 7e; Figure S16a, Supporting Information). However, HE‐LaNb_3_O_9_ offers a larger reversible capacity of 140.2 mAh g^−1^ with a lower initial Coulombic efficiency of 66.7% at 0.1C than that at 25 °C, and both values are larger than that of LaNb_3_O_9_ (98.3 mAh g^−1^ and 52.7%, Figure S16b, Supporting Information). These results can be ascribed to the fact that thicker SEI films form on HE‐LaNb_3_O_9_ at 60 °C during the initial lithiation process (Figure S10a,c, Supporting Information) but its SEI films are thinner than that of LaNb_3_O_9_ (Figure S10c,d, Supporting Information), which are in good agreement with the CV curves (Figure 3e,f). When the current rate is increased to 50C, HE‐LaNb_3_O_9_ retains a larger capacity of 97.2 mA g^−1^ with a significantly larger 50C versus 0.1C capacity ratio of 69.3% than that at 25 °C (Figure 7g), revealing its significantly higher rate capability at the high temperature. Importantly, this rate capability of HE‐LaNb_3_O_9_ is even higher than that of LaNb_3_O_9_ with a 50C versus 0.1C capacity ratio of 63.2%, which is undoubtedly due to the faster high‐temperature Li^+^ diffusivity of HE‐LaNb_3_O_9_ than that of LaNb_3_O_9_. In addition, HE‐LaNb_3_O_9_ still exhibits better cyclability (94.7% capacity retention at 50C after 1000 cycles, Figure 7h) than LaNb_3_O_9_ (90.9% capacity retention at 0.5C after 1000 cycles). In summary, the high‐entropy modification improves the reversible capacity, cyclability, and high‐temperature performance of LaNb_3_O_9_.

To demonstrate the good practicability of HE‐LaNb_3_O_9_, a LiFePO_4_/HE‐LaNb_3_O_9_ full cell is assembled (Figure 7i), from that an initial discharge capacity of 123.9 mAh g^−1^ and average voltage of 1.94 V are obtained (Figure 7j). The voltage can be significantly increased once a high‐voltage cathode material (such as spinel LiNi_0.5_Mn_1.5_O_4_) is used. This full cell retains 117.1, 109.8, 100.2, and 78.0 mAh g^−1^ at 0.5, 1, 2, and 5C, respectively (Figure 7k), showing high rate capability with a 5C versus 0.1C capacity ratio of 62.9%. Additionally, it exhibits superior cyclability with capacity retention of 92.5% at 2C and 95.5% at 5C over 1000 cycles (Figure 7l).

## 3 Conclusion

In this study, we prepare a high‐entropy La_1/4_Ce_1/4_Pr_1/4_Nd_1/4_Nb_3_O_9_ by substituting different elements in LaNb_3_O_9_, and systematically study the effects of the four core factors of high entropy on HEMs for electrochemical energy storage. First, the fact that pure HE‐LaNb_3_O_9_ (1200 °C) can be obtained at lower‐temperatures than that of LaNb_3_O_9_ is attributed to the high‐entropy effects. Second, due to the lattice distortion in HE‐LaNb_3_O_9_, its maximum unit‐cell‐volume change (−1.36%) is decreased, resulting in its better cyclability (95.1% capacity retention after 2000 cycles at 20C). Third, the Li^+^ diffusivity of HE‐LaNb_3_O_9_ (2.35 × 10^−12^ cm^2^ s^−1^) at 25 °C is limited by the restrained diffusion rooted in the lattice distortion, leading to the rate‐capability decay (50C vs 0.1C capacity ratio of 55.2%). However, the alleviated lattice distortion in HE‐LaNb_3_O_9_ at 60 °C facilitates its Li^+^ diffusivity (6.63 × 10^−12^ cm^2^ s^−1^), greatly contributing to its higher high‐temperature rate capability (50C vs 0.1C capacity ratio of 69.3%) than that of LaNb_3_O_9_. Finally, the multi‐ion interaction of HE‐LaNb_3_O_9_ rooted in the cocktail effects allows its significantly higher electronic conductivity (3.6 × 10^−7^ S cm^−1^), better electrochemical activity, more intensive Nb^5+^ ↔ Nb^3+^ redox reaction, and larger reversible capacity (136.2 mAh g^−1^ at 0.1C). Based on the understanding of the four core factors of high entropy on HEMs, various methods for improving the electrochemical energy‐storage properties can be explored in the future.

## 4 Experimental Section

### Material Fabrications

The micron‐sized La_1/4_Ce_1/4_Pr_1/4_Nd_1/4_Nb_3_O_9_ (HE‐LaNb_3_O_9_) and LaNb_3_O_9_ particles were fabricated by solid‐state reaction. The fabrication of HE‐LaNb_3_O_9_ used La_2_O_3_ (Macklin, 99.99%), CeCl_3_ (Macklin, 99.99%), Pr_2_O_3_ (Macklin, 99.9%), Nd_2_O_3_ (Aladdin, 99.99%), and Nb_2_O_5_ (Macklin, 99.9%) in a molar ratio of 1/4:1/4:1/4:1/4:3 as raw materials. The fabrication of LaNb_3_O_9_ used La_2_O_3_ (Macklin, 99.99%) and Nb_2_O_5_ (Macklin, 99.9%) in a molar ratio of 1:3 as raw materials. They were ball‐milled by using a ball‐milling machine (SPEX 8000 M) for 1 h. The resulting HE‐LaNb_3_O_9_ and LaNb_3_O_9_ mixtures were respectively calcined in air at 1200 and 1300 °C for 5 h.

### Material Characterizations

The powder X‐ray diffraction (XRD) patterns were recorded on an X‐ray diffractometer (Rigaku Smart Lab) with Cu‐K*α* radiation. The Rietveld refinements were conducted by the General Structure Analysis System (GSAS) program.^[^ ^20^ ^]^ JEOL JSM‐7800F field emission scanning electron microscopy (FESEM) equipped with OXFORD X‐Max energy dispersive spectroscopy (EDS) and JEOL JEM‐2100Plus high‐resolution transmission electron microscopy (HRTEM) were employed to systematically examine the morphologies, sizes, and structures of the particles. An ASAP 2460 surface area analyzer was used to examine the specific surface areas, in which the Branauer–Emmett–Teller (BET) method was used. A SolidSpec 3600 Plus UV–vis spectrophotometer was employed to measure the absorbance of the materials. PHI5000 Versaprobe X‐ray photoelectron spectroscopy (XPS) was used to analyze the compositions and elemental valences. The electronic‐conductivity test of a compact HE‐LaNb_3_O_9_ (or LaNb_3_O_9_) pellet was performed through a two‐probe direct current method. The pellet, having with a diameter of 10 mm and thickness of 1 mm, was constructed through pressing the HE‐LaNb_3_O_9_ (or LaNb_3_O_9_) powder in a home‐designed module. An inductively coupled plasma atomic emission spectrometer (ICP‐AES, Agilent ICPOES730) was employed to determine the exact molar ratio of the cations in HE‐LaNb_3_O_9_.

### Electrochemical Tests

The working electrodes consisted of HE‐LaNb_3_O_9_ (or LaNb_3_O_9_), Super‐P carbon conductive additive, and polyvinylidene fluoride binder in a weight ratio of 7:2:1 (or 8:1:1 for large electrode loading). These components were mixed in 1‐methyl‐2‐pyrrolidinone to form a slurry, which was coated on Cu foils. After being fully dried, the Cu foils were cut into circular electrodes with a diameter of 12 mm and active‐material loading of ≈1.1 (or ≈6.4) mg cm^−2^, which serviced as the cathodes of HE‐LaNb_3_O_9_/Li (or LaNb_3_O_9_/Li) half cells (CR2016 type) assembled in an Ar‐filled glove box. For all electrochemical measurements, the electrolyte was 1 m LiPF_6_ dissolving in the mixture of diethylene carbonate, ethylene carbonate, and dimethyl carbonate in a 1:1:1 volume ratio, and the separators were Celgard 2325 microporous polypropylene films. For the LiFePO_4_/HE‐LaNb_3_O_9_ full cell (CR2025 type), a HE‐LaNb_3_O_9_ electrode was employed as the anode, and a LiFePO_4_ electrode (70 wt.% LiFePO_4_, 20 wt.% conductive additive, and 10 wt.% binder) was selected as the cathode, with a LiFePO_4_: HE‐LaNb_3_O_9_ weight ratio of ≈1.5:1.

The galvanostatic charge–discharge (GCD) tests and galvanostatic intermittent titration technique (GITT) experiments were performed on a Neware CT‐3008 multichannel battery tester at room (25 °C) and high (60 °C) temperatures controlled in a temperature‐variable cryostat system (Linpin LRHS‐101C). The current density of 1C corresponds to the theoretical capacity, i.e., 285 mA g^−1^ for HE‐LaNb_3_O_9_ and 286 mA g^−1^ for LaNb_3_O_9_. A Gamry 1010E electrochemical workstation was employed to record cyclic voltammetry (CV) curves at different sweep rates. The voltage ranges for testing the half and full cells were 0.8–3.0 V versus Li/Li^+^ and 1.0–2.6 V, respectively.

### In Situ Examinations

The in situ XRD experiments within 10–50° were carried out based on commercial modules (Scistar LIB‐XRD for the room‐temperature tests and Scistar LIB‐LHTXRD‐LN for the high‐temperature tests), equipped with a Be window and current collector per module. The high temperature at 60 °C was controlled using a specially designed temperature‐control unit and a polyetheretherketone (PEEK) dome.^[^ ^21^ ^]^ All the recorded in situ XRD patterns were refined using the Rietveld method.

The in situ TEM examinations were performed using a specially designed electrochemical holder.^[^ ^22^ ^]^ HE‐LaNb_3_O_9_‐loaded W rod, Li‐loaded W rod, and Li_2_O naturally coated on Li‐metal surfaces were used as the working electrode, counter electrode, and solid electrolyte, respectively. When the two electrodes were in contact and a proper external voltage was applied, the Li → Li_2_O → HE‐LaNb_3_O_9_ lithiation was observed.

## Conflict of Interest

The authors declare no conflict of interest.

## Supporting information



Supporting Information

Supplementary Video 1

## Data Availability

The data that support the findings of this study are available from the corresponding author upon reasonable request.
