# Supplementary material for: How Do the Four Core Factors of High Entropy Affect the Electrochemical Properties of Energy‐Storage Materials?
Source: Adv Sci (Weinh). 2024 Nov 4;12(1):2411291. doi: 10.1002/advs.202411291 (PMC11714241; doi:10.1002/advs.202411291)
Supplement: Supplementary file 1 — Supporting Information [file ADVS-12-2411291-s001.docx]

Supporting Information

How do the Four Core Factors of High Entropy Affect the Electrochemical Properties of Energy-Storage Materials?

Wenze Wang, Qian Zhang, Liting Yang, Guisheng Liang, Xuhui Xiong, Yifeng Cheng, Limin Wu*, Chunfu Lin*, and Renchao Che*


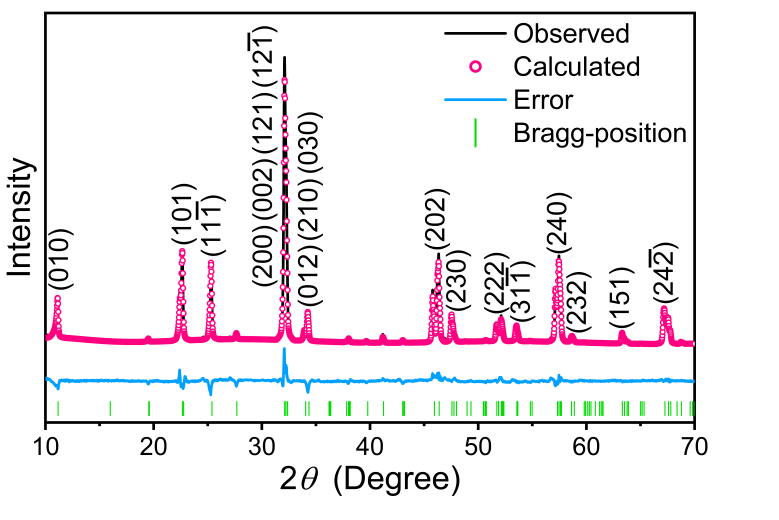


**Figure S1.** Rietveld-refined XRD pattern of LaNb_3_O_9_ (main peaks are labelled).


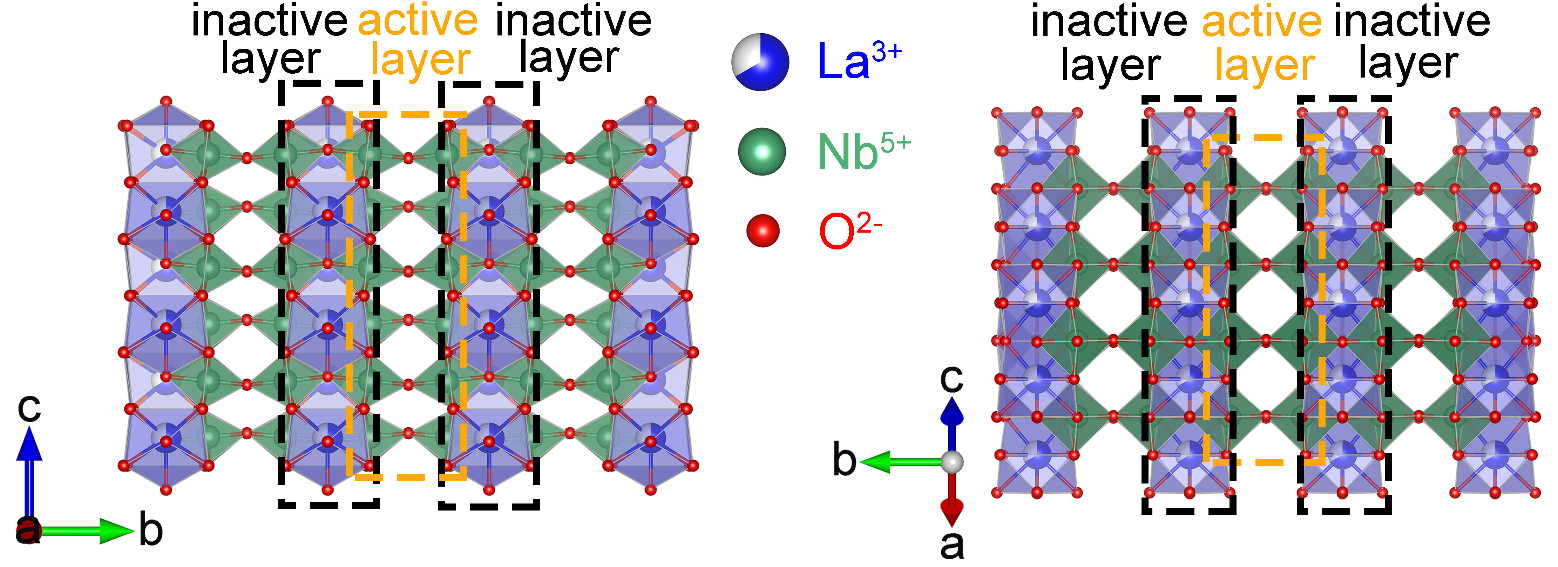


**Figure S2.** Schematic crystal structure of LaNb_3_O_9_.

**Calculation of molar configurational entropy**

The molar configurational entropy can be calculated based on **Equation S1**:^[S1,S2]^

$${\Delta S}_{\mathrm{config}}=-R\left[ \left( \sum_{i=1}^{n} x_{i}{\ln x}_{i} \right)_{\mathrm{cation}-\mathrm{site}}+\left( \sum_{j=1}^{m} x_{j}\ln x_{j} \right)_{anion-site} \right] (S1)$$

In this equation, $\Delta$*S*_config_ describes the configurational entropy; *R* is the ideal gas constant; *x_i_* and *x_j_* represent the mole fraction of the *i*_th_ and *j*_th_ component in the cation and anion sites, respectively; *n* and *m* correspond to the number of elements in the cation and anion sites, respectively.


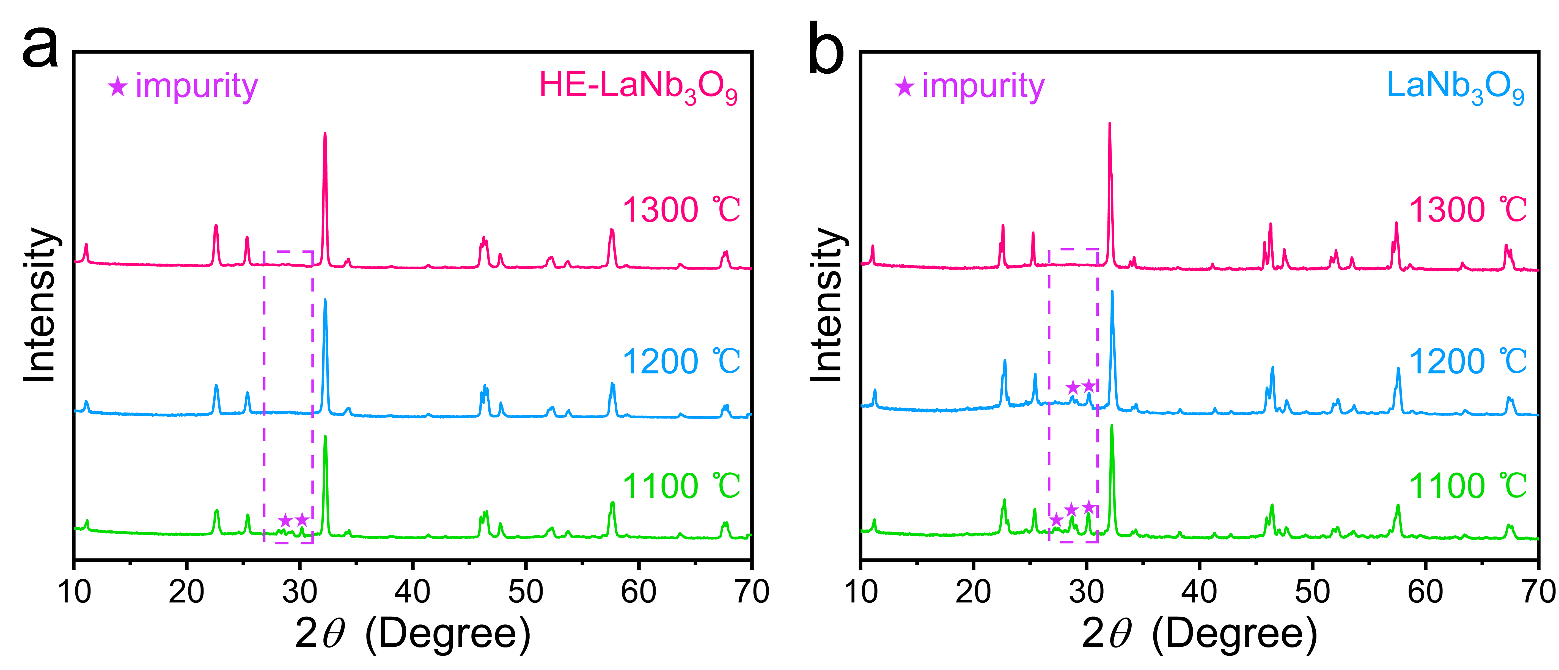


**Figure S3.** Powder XRD patterns of a) HE-LaNb_3_O_9_ and b) LaNb_3_O_9_ at 1100, 1200, and 1300 ℃.

**
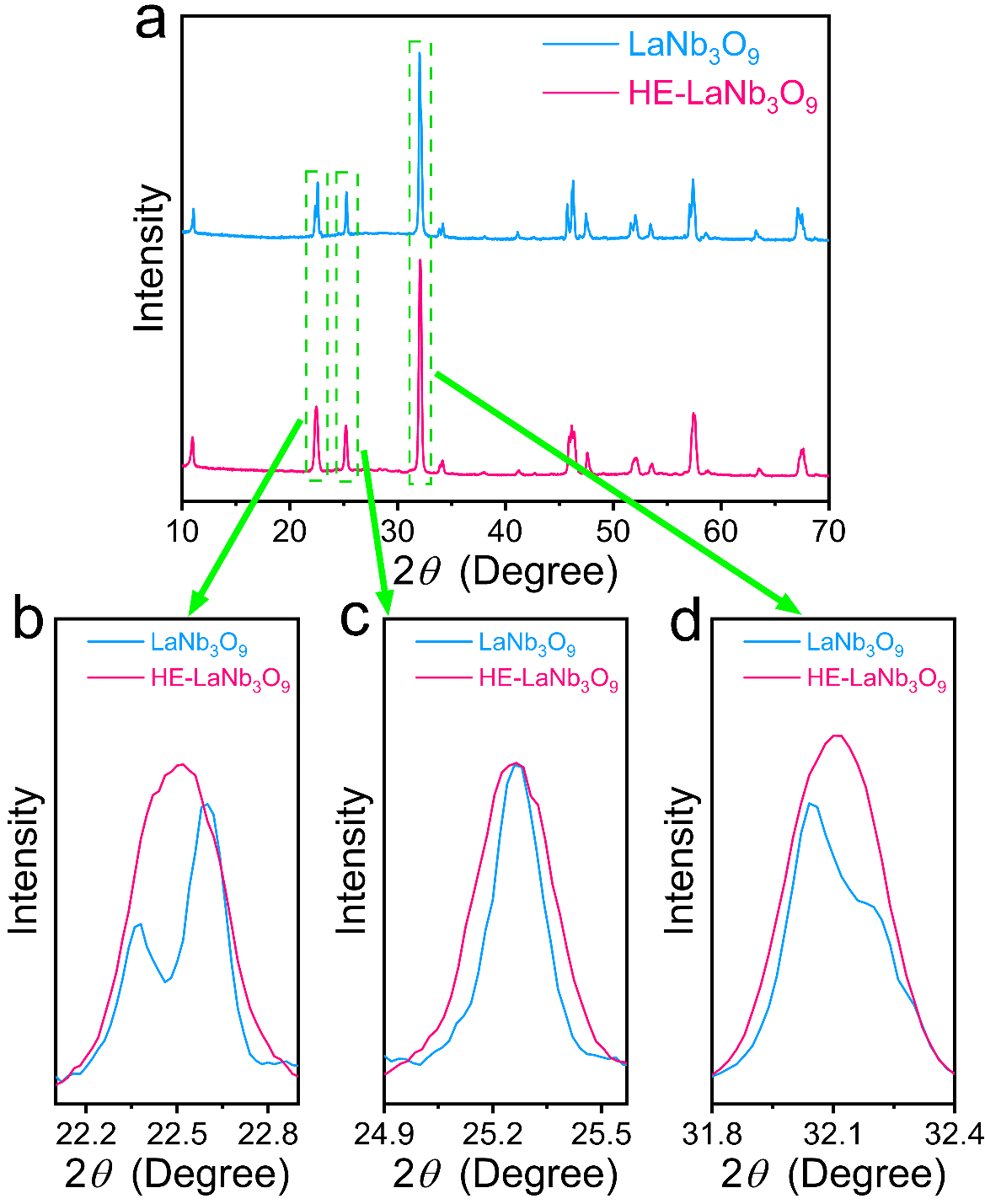
**

**Figure S4.** a) Powder XRD patterns of HE-LaNb_3_O_9_ and LaNb_3_O_9_ at 1300 ℃. Powder XRD patterns of HE-LaNb_3_O_9_ and LaNb_3_O_9_ at 1300 ℃ enlarged within b) 22.1–22.9°, c) 24.9–25.6°, and d) 31.8–32.4°.


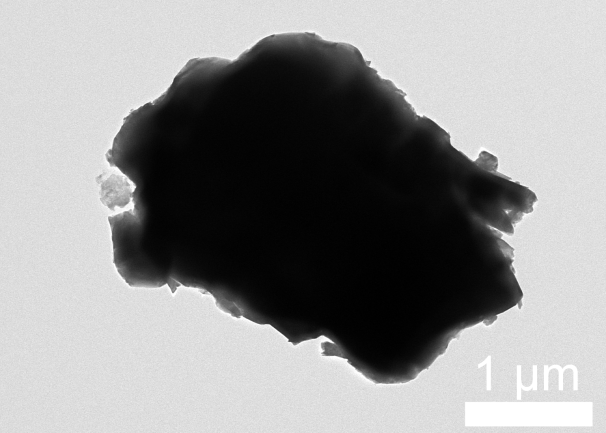


**Figure S5.** TEM image of HE-LaNb_3_O_9_.


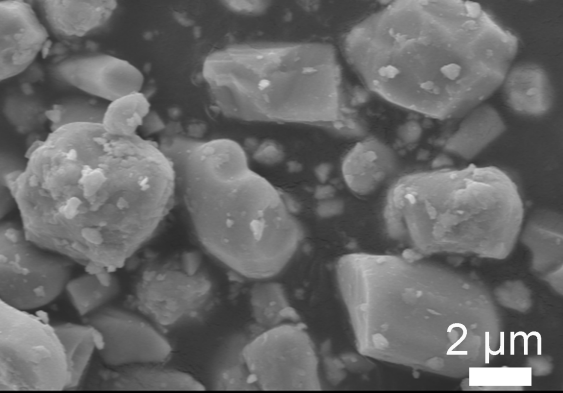


**Figure S6.** FESEM image of LaNb_3_O_9_.


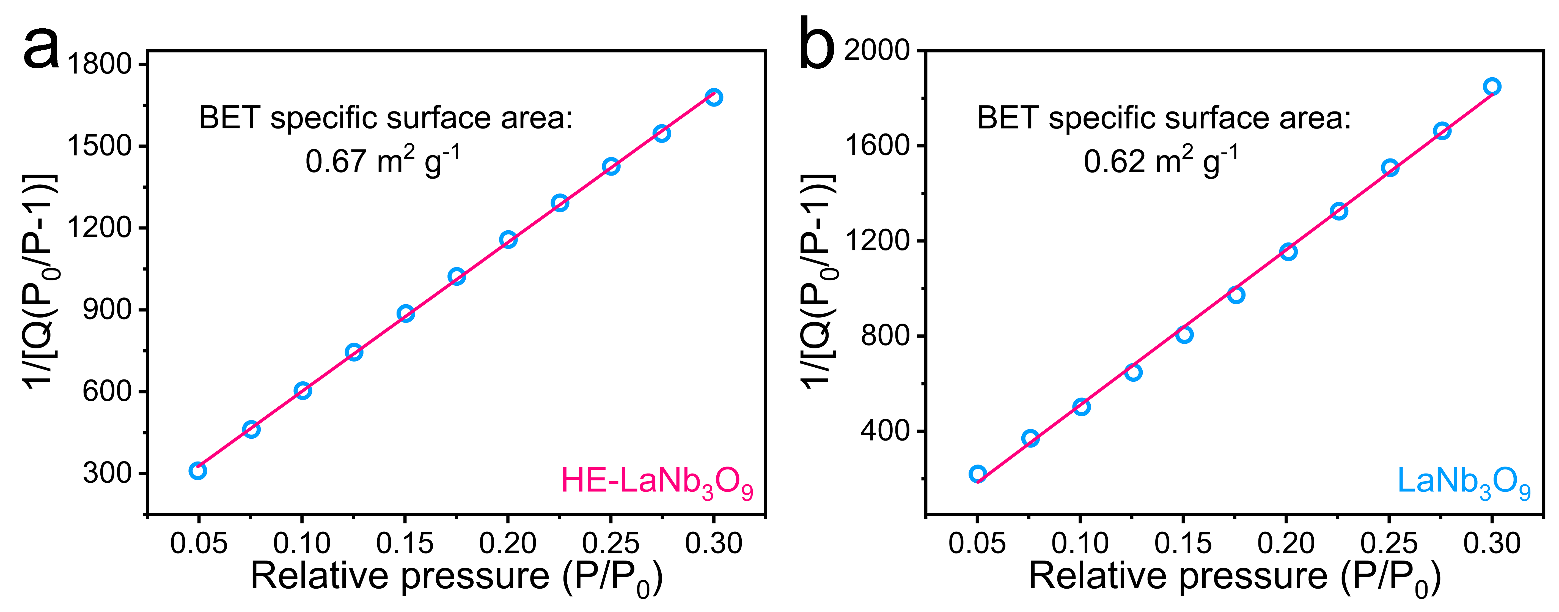


**Figure S7.** BET specific surface area plots of a) HE-LaNb_3_O_9_ and b) LaNb_3_O_9_.


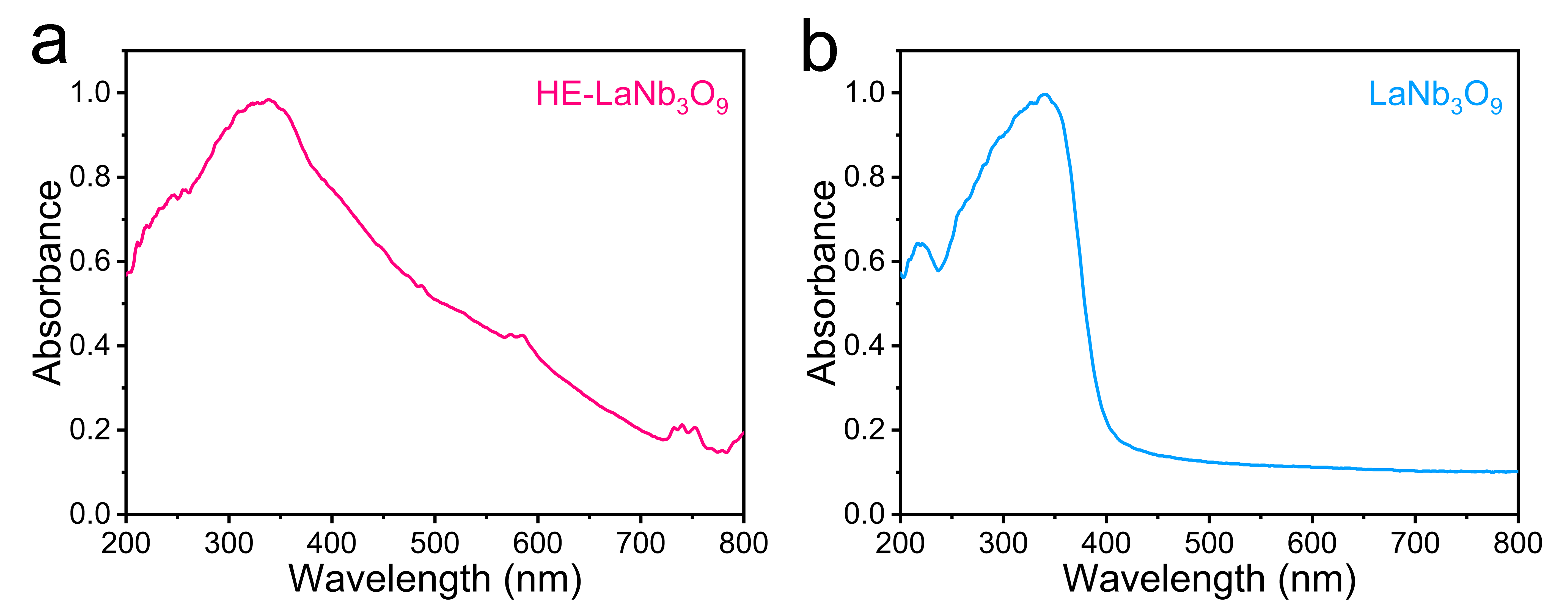


**Figure S8.** UV–vis absorption spectra of a) HE-LaNb_3_O_9_ and b) LaNb_3_O_9_.

**Description of Equation S2**

According to the Tauc plot method, the following **Equation S2** is used to calculate the band gap energy:^[S3]^

(*αhv*)*^n^* = *K*(*hv −* *E*_g_) (S2)

where *α* is the absorption coefficient, *n* is a constant, *K* is the energy independence coefficient, *hv* is the incident photon energy, and *E*_g_ is the band gap energy. The band gap energies of HE-LaNb_3_O_9_ and LaNb_3_O_9_ are calculated to be 2.28 and 3.21 eV, respectively.


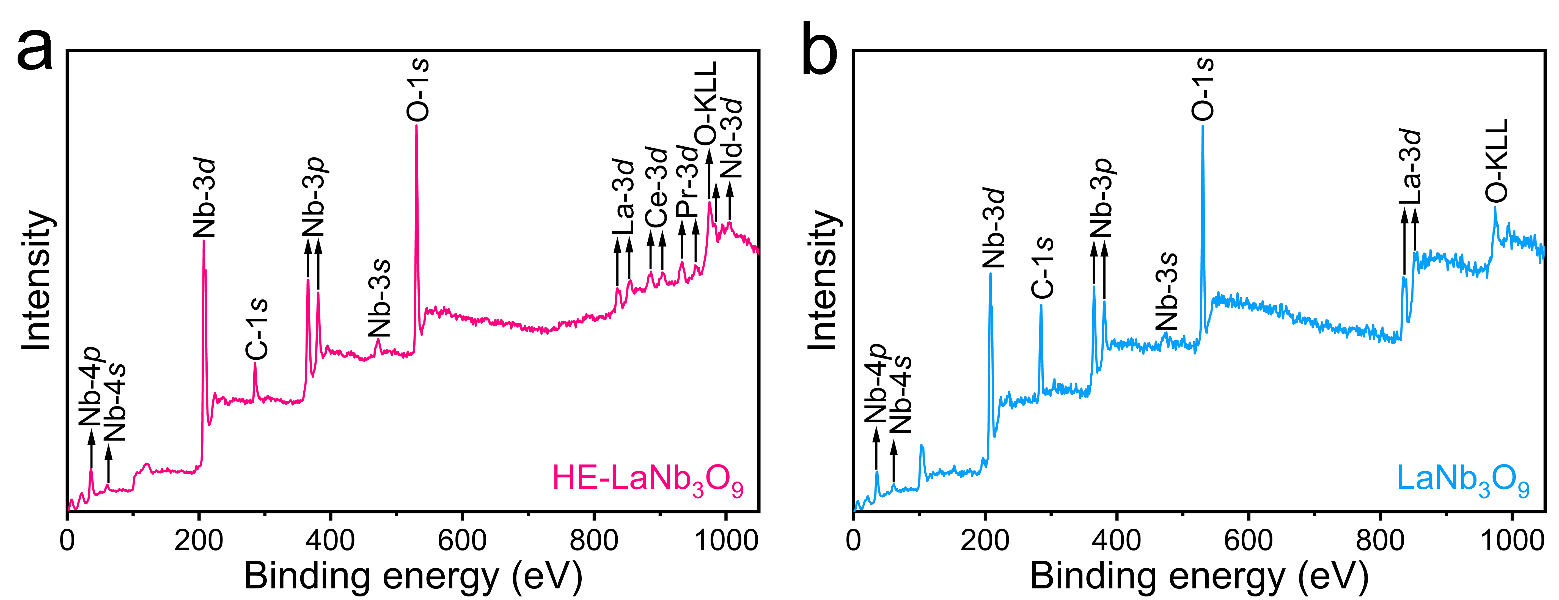


**Figure S9.** XPS survey spectra of a) HE-LaNb_3_O_9_ and b) LaNb_3_O_9_.


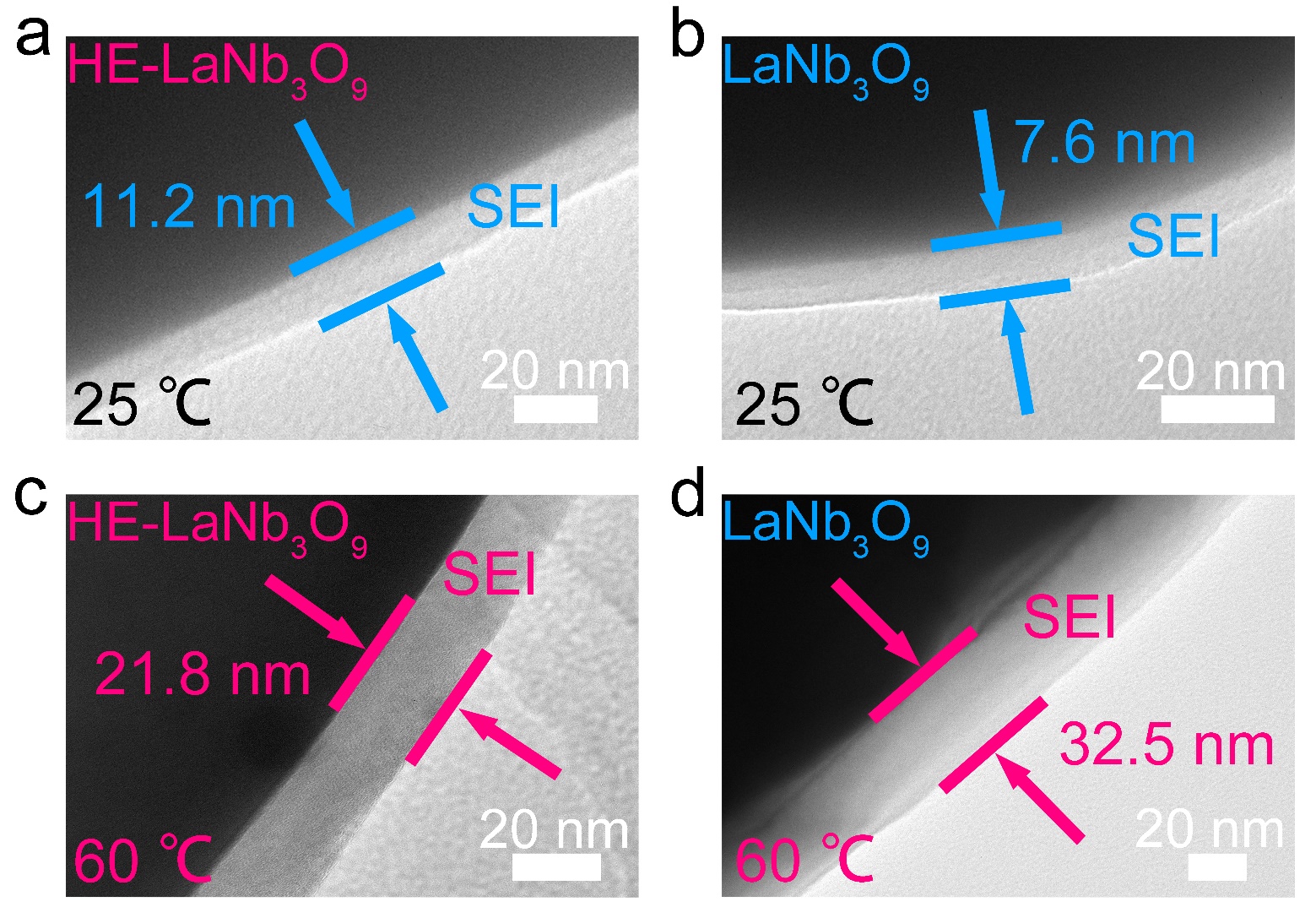


**Figure S10.** TEM images of a) HE-LaNb_3_O_9_ and b) LaNb_3_O_9_ samples showing SEI films after first lithiation at 25 ℃. TEM images of c) HE-LaNb_3_O_9_ and d) LaNb_3_O_9_ samples showing SEI films after first lithiation at 60 ℃.


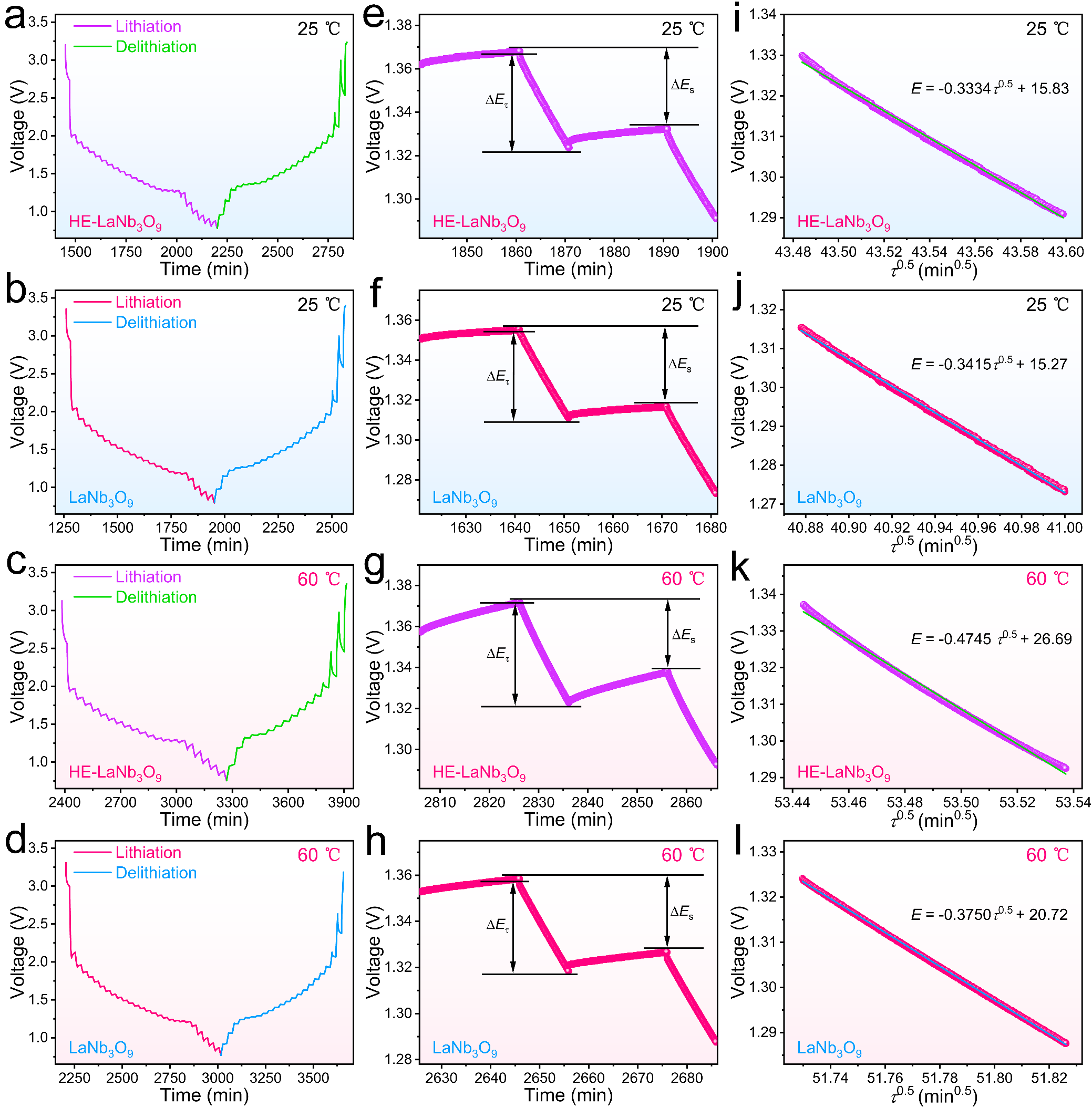


**Figure S11.** Second-cycle GITT lithiation–delithiation curves of a) HE-LaNb_3_O_9_ and b) LaNb_3_O_9_ at 0.1C and 25 ℃. Second-cycle GITT lithiation–delithiation curves of c) HE-LaNb_3_O_9_ and d) LaNb_3_O_9_ at 0.1C and 60 ℃. *E* *vs.* *t* curves for a single step in GITT experiment of e) HE-LaNb_3_O_9_ and f) LaNb_3_O_9_ at 25 ℃. *E* *vs.* *t* curves for a single step in GITT experiment of g) HE-LaNb_3_O_9_ and h) LaNb_3_O_9_ at 60 ℃. Linear behavior of *E* *vs.* *τ*^0.5^ relationship during a typical titration in i) HE-LaNb_3_O_9_ and j) LaNb_3_O_9_ at 25 ℃. Linear behavior of *E* *vs.* *τ*^0.5^ relationship during a typical titration in k) HE-LaNb_3_O_9_ and l) LaNb_3_O_9_ at 60 ℃.

**Calculation of apparent Li^+^ diffusion coefficients of HE-LaNb_3_O_9_ and LaNb_3_O_9_ by GITT**

The GITT experiments are employed to investigate the Li^+^ diffusion behavior in the HE-LaNb_3_O_9_ and LaNb_3_O_9_ lattices at different temperature. **Figure S11a** exhibits the second-cycle GITT curves of the HE-LaNb_3_O_9_/Li half cells at 25 °C. A typical single step of GITT is clearly seen from **Figure S11e**. On the basis of the Fick’s second law, the apparent Li^+^ diffusion coefficients (*D*_Li_) of HE-LaNb_3_O_9_ can be determined by using **Equation S3**:^[S4]^

$D_{\mathrm{Li}}=\frac{4}{\pi\tau}\left( \frac{m_{B}V_{m}}{M_{B}S} \right)^{2}\left( \frac{{\Delta E}_{s}}{\tau\left( {{dE}_{\tau}}/{d\sqrt{\tau}} \right)} \right)^{2}$ $\left( \tau\leq\frac{L^{2}}{D_{\mathrm{Li}}} \right)$ (S3)

where, *M*_B_ is the molar mass of HE-LaNb_3_O_9_, *V*_m_ is the molar volume of HE-LaNb_3_O_9_, *m*_B_ is the mass of HE-LaNb_3_O_9_, *S* is the electrode surface area, *τ* is the pulse duration time, *L* is the electrode thickness, and Δ*E*_s_ and Δ*E*_τ_ respectively represent the change in the equilibrium potential and the change in potential during the current pulse, which can be gained from the GITT curves (**Figure S11e**). As the potential during a single titration delivers a linear relationship with *τ*^0.5^ (**Figure S11i**), **Equation S3** can be simplified as **Equation S4**:

$D_{\mathrm{Li}}=\frac{4}{\pi\tau}\left( \frac{m_{B}V_{m}}{M_{B}S} \right)^{2}\left( \frac{\Delta E_{s}}{{\Delta E}_{\tau}} \right)^{2}$ $\left( \tau\leq\frac{L^{2}}{D_{Li}} \right)$ (S4)

Based on **Equation S4**, the apparent Li^+^ diffusion coefficients of HE-LaNb_3_O_9_ at 25 °C during different states of discharge/charge are obtained, and displayed in **Figure 3i**. Using the same GITT method, the Li^+^ diffusivity of LaNb_3_O_9_ at 25 ℃ is also analyzed and illustrated in **Figure 3i**, and that of the two materials at 60 ℃ is demonstrated in **Figure 3j**.


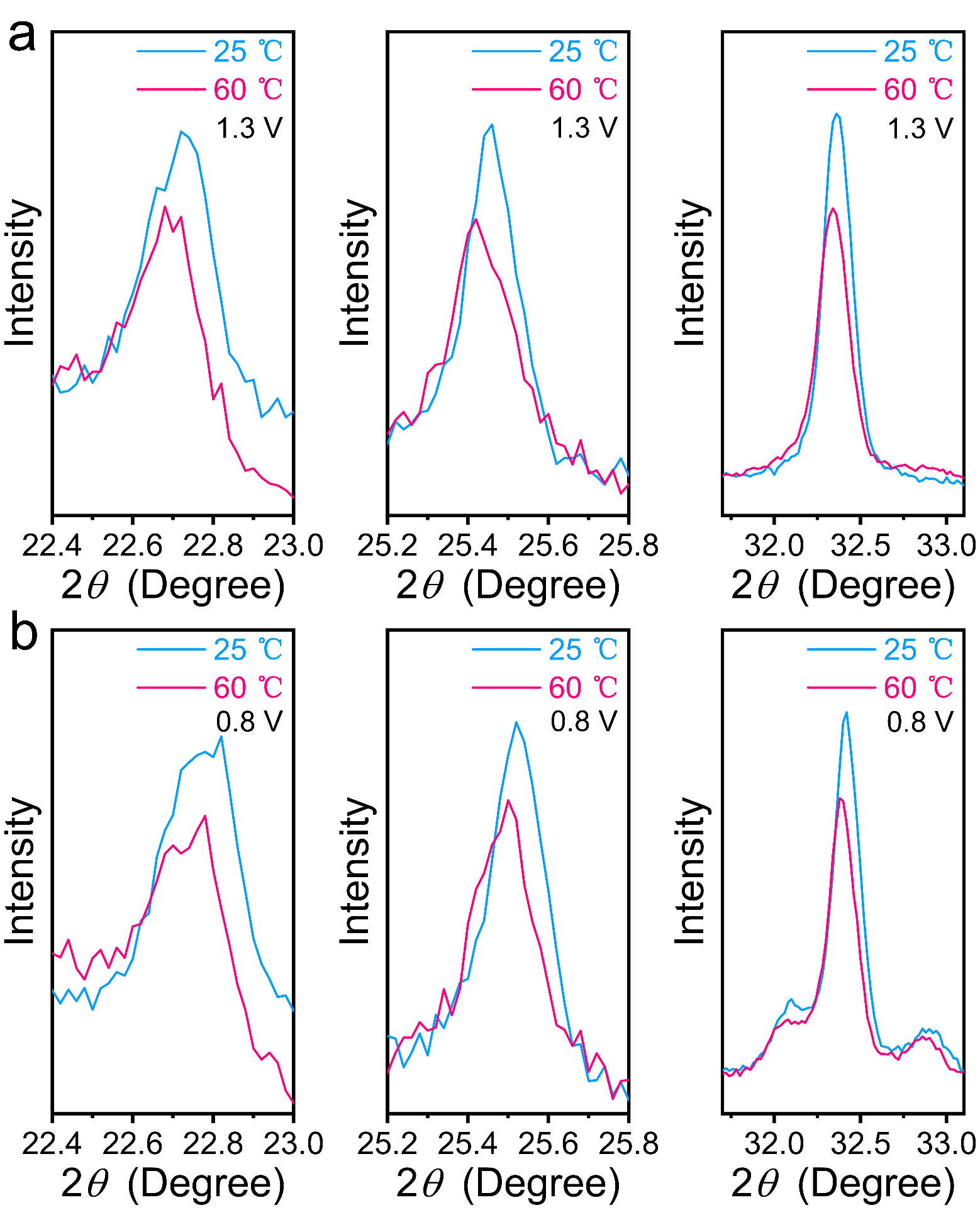


**Figure S12.** *In-situ* XRD peaks of HE-LaNb_3_O_9_ at 25 and 60 ℃ when first discharge to a) 1.3 and b) 0.8 V.


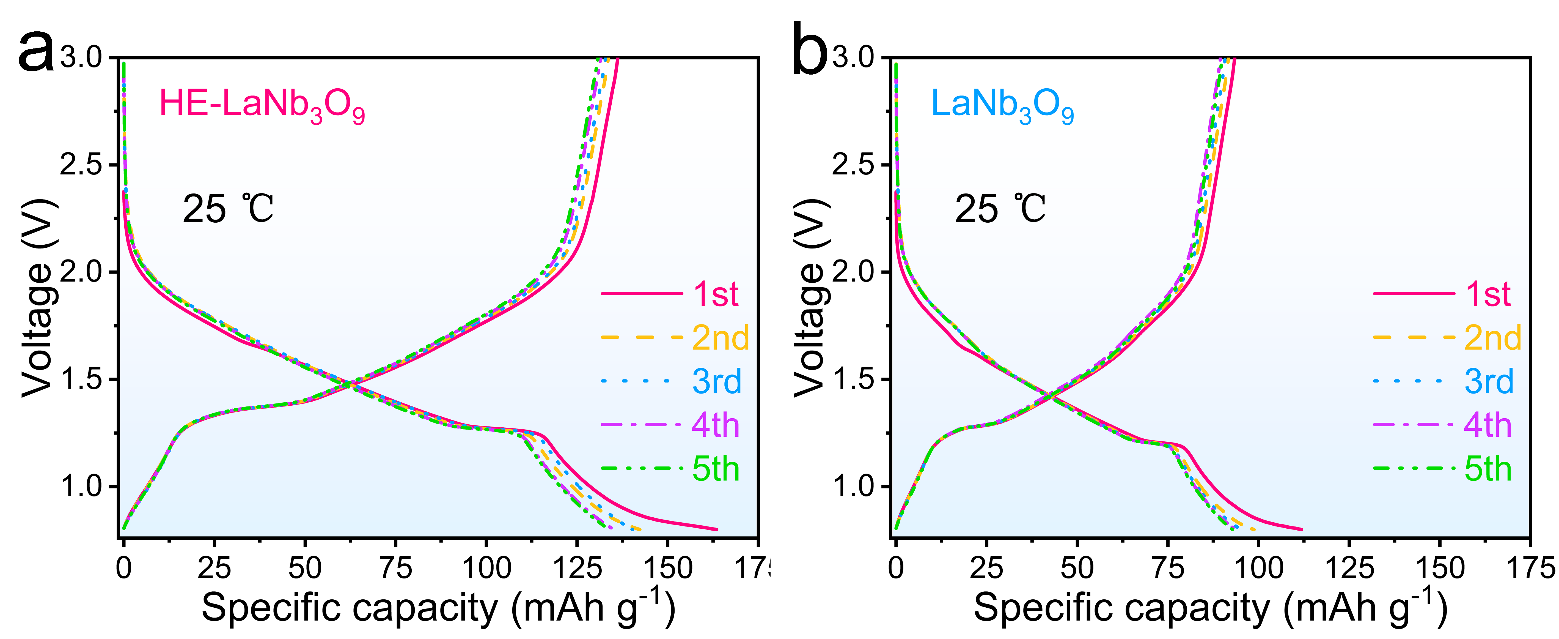


**Figure S13.** GCD curves of a) HE-LaNb_3_O_9_/Li and b) LaNb_3_O_9_/Li half cells for initial five cycles at 0.1C and 25 ℃.


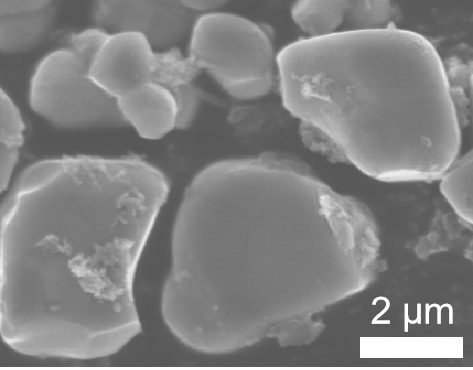


**Figure S14.** FESEM image of HE-LaNb_3_O_9_ after long-term cycling 2000 cycles at 20C.


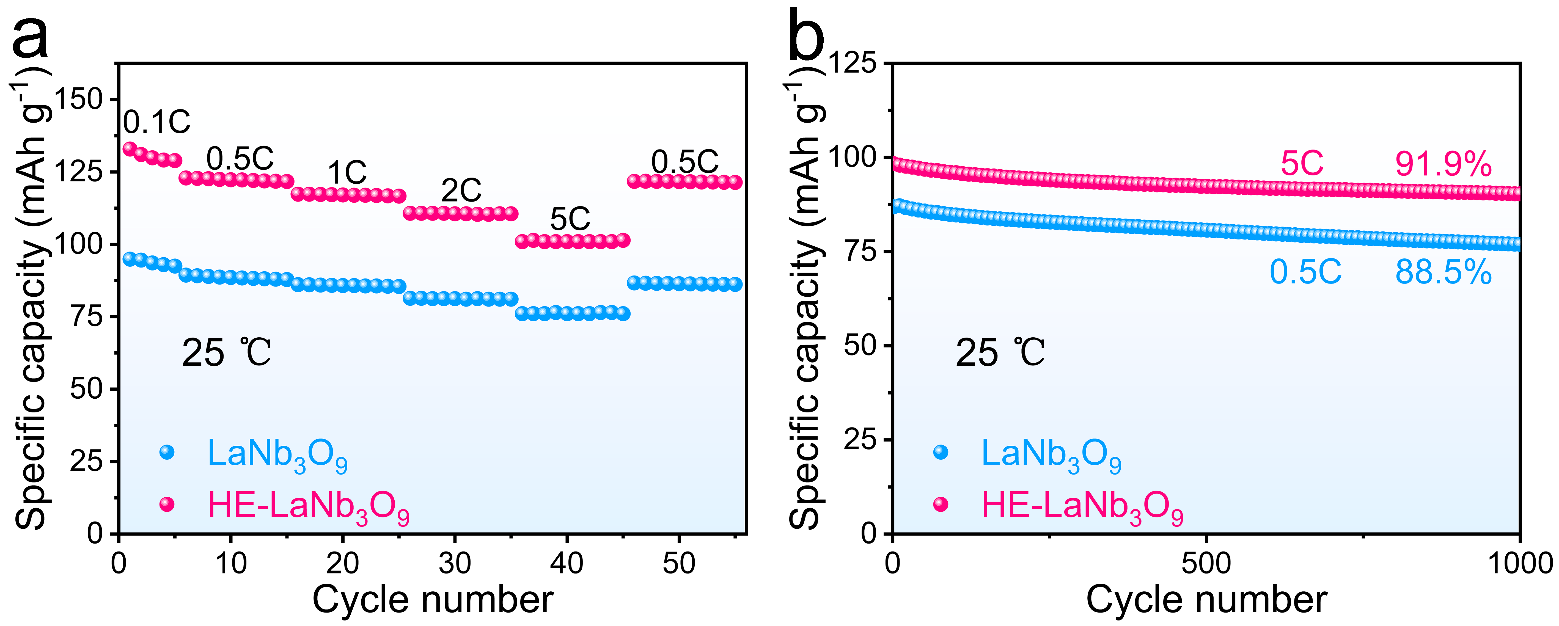


**Figure S15.** Electrochemical properties of HE-LaNb_3_O_9_ and LaNb_3_O_9_ with high active material content (80 wt%) and large active-material loading (~6.4 mg cm^−2^) at 25 ℃: a) rate capability, and b) cyclability at 10C over 1000 cycles.


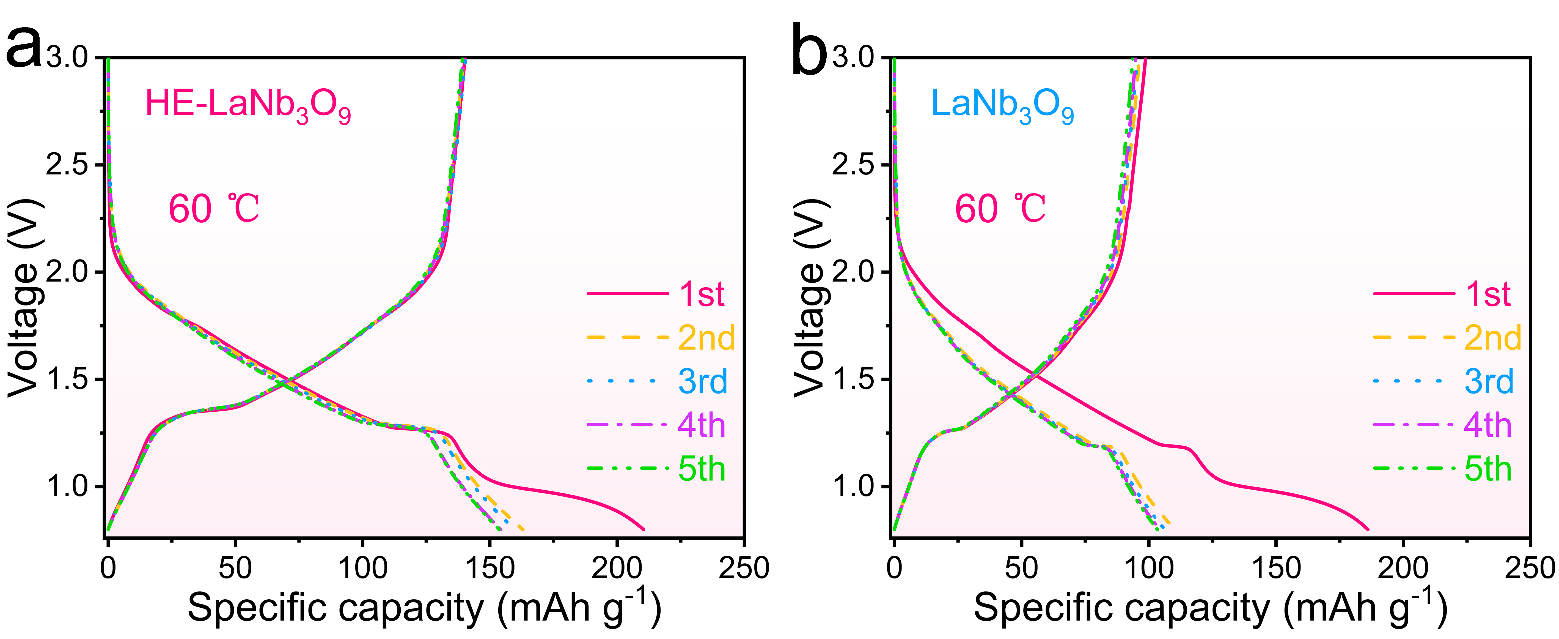


**Figure S16.** GCD curves of a) HE-LaNb_3_O_9_/Li and b) LaNb_3_O_9_/Li half cells for initial five cycles at 0.1C and 60 ℃.

**Table S1.** Fractional atomic parameters of HE-LaNb_3_O_9_ with *P2/m* space group.

| atom | site | *x* | *y* | *z* | occupancy |
| --- | --- | --- | --- | --- | --- |
| La | 2*m* | 0.2468 | 0 | 0.7447 | 0.1667 |
| Ce | 2*m* | 0.2468 | 0 | 0.7447 | 0.1667 |
| Pr | 2*m* | 0.2468 | 0 | 0.7447 | 0.1667 |
| Nd | 2*m* | 0.2468 | 0 | 0.7447 | 0.1667 |
| Nb | 4*o* | 0.2508 | 0.2611 | 0.2483 | 1 |
| O1 | 2*n* | 0.2309 | 0.5 | 0.3468 | 1 |
| O2 | 2*m* | 0.2731 | 0 | 0.2902 | 1 |
| O3 | 2*l* | 0.5 | 0.2277 | 0.5 | 1 |
| O4 | 2*k* | 0 | 0.2178 | 0.5 | 1 |
| O5 | 2*j* | 0.5 | 0.2489 | 0 | 1 |
| O6 | 2*i* | 0 | 0.3227 | 0 | 1 |

**Table S2.** Fractional atomic parameters of LaNb_3_O_9_ with *P2/m* space group.

| atom | site | *x* | *y* | *z* | occupancy |
| --- | --- | --- | --- | --- | --- |
| La | 2*m* | 0.2483 | 0 | 0.7512 | 0.6667 |
| Nb | 4*o* | 0.2493 | 0.2601 | 0.2495 | 1 |
| O1 | 2*n* | 0.2786 | 0.5 | 0.2858 | 1 |
| O2 | 2*m* | 0.2279 | 0 | 0.2264 | 1 |
| O3 | 2*l* | 0.5 | 0.2239 | 0.5 | 1 |
| O4 | 2*k* | 0 | 0.2375 | 0.5 | 1 |
| O5 | 2*j* | 0.5 | 0.2377 | 0 | 1 |
| O6 | 2*i* | 0 | 0.4264 | 0 | 1 |

**References**

[S1] A. Sarkar, L. Velasco, D. Wang, Q. Wang, G. Talasila, L. de Biasi, C. Kübel, T. Brezesinski, S.S. Bhattacharya, H. Hahn, B. Breitung, High entropy oxides for reversible energy storage, Nat. Commun. 9 (2018) 3400.

[S2] A. Sarkar, B. Breitung, H. Hahn, High entropy oxides: The role of entropy, enthalpy and synergy, Scr. Mater. 187 (2020) 43–48.

[S3] P. George, P. Chowdhury, Complex dielectrics transformation of UV–vis diffused reflectance spectra for estimating optical band-gap energies and materials classification, Analyst 144 (2019) 3005–3012.

[S3] A.J. Bard, L.R. Faulkner, Electrochemical Methods: Fundamentals and Applications, second ed., Wiley, New York, 2001.
